# Circulating autoantibodies to alpha-enolase (ENO1) and far upstream element-binding protein 1 (FUBP1) are negative prognostic factors for pancreatic cancer patient survival

**DOI:** 10.1007/s10238-023-01236-5

**Published:** 2023-11-01

**Authors:** Claudia Curcio, Tiziana Rosso, Silvia Brugiapaglia, Giorgia Guadagnin, Daniele Giordano, Bruno Castellino, Maria Antonietta Satolli, Rosella Spadi, Donata Campra, Francesco Moro, Mauro Giulio Papotti, Luca Bertero, Paola Cassoni, Claudio De Angelis, Serena Langella, Alessandro Ferrero, Serena Armentano, Giovanna Bellotti, Elisabetta Fenocchio, Annamaria Nuzzo, Giovannino Ciccone, Francesco Novelli

**Affiliations:** 1 Laboratory of Tumor Immunology, Department of Molecular Biotechnology and Health Sciences, University of Torino, Turin, Italy; 2 ENOAPA Biobank, SSD Banche Tessuti E Bioconservatorio, AOU Città Della Salute E Della Scienza Di Torino, Turin, Italy; 3 Unit of Clinical Epidemiology, AOU Città Della Salute E Della Scienza Di Torino and CPO Piemonte, Turin, Italy; 4 Centro Oncologico Ematologico Subalpino, AOU Città Della Salute E Della Scienza Di Torino, Turin, Italy; 5 SC Chirurgia Generale d’urgenza E Pronto Soccorso, AOU Città Della Salute E Della Scienza Di Torino, Turin, Italy; 6 SC Chirurgia Generale U2, AOU Città Della Salute E Della Scienza Di Torino, Turin, Italy; 7 Pathology Unit, Department of Medical Sciences, University of Torino, AOU Città Della Salute E Della Scienza Di Torino, Turin, Italy; 8 SCDU Gastroenterology U, AOU Città Della Salute E Della Scienza Di Torino, Turin, Italy; 9 General Surgery and Oncology, Ordine Mauriziano Di Torino, Turin, Italy; 10 Oncology Department, SS. Antonio E Biagio C. Arrigo Di Alessandria, Alessandria, Italy; 11 Candiolo Cancer Institute, FPO–IRCCS, Candiolo, Turin, Italy; 12 Department of Molecular Biotechnology and Health Sciences, Molecular Biotechnology Center, Piazza Nizza 44B, Turin, Italy

**Keywords:** Pancreatic ductal adenocarcinoma, Alpha-enolase, Far upstream element-binding protein 1, Circulating autoantibodies

## Abstract

Pancreatic ductal adenocarcinoma (PDA) has a dismal prognosis due to a lack of early diagnostic markers and effective therapy. In PDA patients, the glycolytic enzyme and plasminogen receptor alpha-enolase (ENO1) and the transcription factor far upstream element-binding protein 1 (FUBP1) are upregulated and elicit the production of autoantibodies (aAb) that discriminate healthy subjects from PDA patients, with the latter mostly directed to post-translational phosphorylated isoforms. Here, the correlation of prognosis with circulating ENO1 and FUBP1aAb, and their protein tissue expression was analyzed in PDA patients. Circulating ENO1 and FUBP1 aAb was analyzed in two cohorts of PDA patients by ELISA (*n* = 470), while tissues expression was observed by immunohistochemistry (*n* = 45). Overall survival (OS) was estimated using the Kaplan–Meier method, while the Cox model was used to estimate the hazard ratios (HR) adjusted for the main prognostic factors. Logistic models were applied to assess associations between death and its risk indicators. All statistical analyses were performed with Stata version 15. Unlike ENO1 aAb, there was a significant correlation between FUBP1 aAb and FUBP1 expression in tumors (*p* = 0.0268). In addition, we found that high ENO1 (*p* = 0.016) and intermediate FUBP1 aAb levels (*p* = 0.013) were unfavorable prognostic factors. Notably, it was found that high anti-FUBP1 aAb level is a good prognostic marker for tail-body PDA (*p* = 0.016). Our results suggest that different levels of circulating aAb to ENO1 and FUBP1 predict a poor outcome in PDA patients and can be used to improve therapeutic strategies.

**Supplementary Information:**

The online version contains supplementary material available at 10.1007/s10238-023-01236-5.

## Background

Pancreatic ductal adenocarcinoma (PDA) is one of the most lethal malignancies, with an extremely poor prognosis and an overall 5-year survival rate of about 7% [1]. To date, surgery is the only treatment that can significantly increase survival of patients [2]. Poor PDA prognosis is related to the absence of early diagnostic markers [3] and the lack of effective therapies that can increase patient survival [4]. In addition, the aggressiveness of PDA calls for urgent strategies for diagnosis and prevention of this tumor.

Autoantibodies (aAb) to different oncogenic proteins in tumor patients have been reported [5–8]. Through a serological proteome approach, we identified alpha-enolase (ENO1) as a key glycolytic enzyme upregulated in PDA [9, 10]. We found that aAb to phosphorylated ENO1 discriminated healthy subjects from PDA patients and usefully complemented the diagnostic performance of serum carbohydrate antigen (CA) 19.9, achieving approximately 95% diagnostic accuracy in both advanced and resectable PDA [10].

We also showed that in PDA patients, circulating anti-far upstream element-binding protein 1 (FUBP1) aAb was higher than in healthy subjects [11]. FUBP1 is highly expressed in different types of cancers [12–19]. When complexed with the far upstream element (FUSE) site that negatively regulates c-Myc expression, FUBP1 promotes tumor growth and glycolysis of cancer cells [19–22]. Unfavorable prognosis of oligodendroglial tumors has been associated with mutation in the FUBP1 gene [23]. FUBP1 deficiency alters cell cycle progression and causes resistance of cells to cell death [24–27]. In PDA cells, FUBP1 promotes cell proliferation, migration and regulates cancer cell immunity by increasing PD-L1 [28, 29]. Knockdown of FUBP1 downregulates the epithelial-to-mesenchymal transition and regulates the TGFβ/Smad signaling cascade [29]. Both FUBP1 and ENO1 expression was upregulated in tumoral tissues compared with adjacent normal tissues and correlated with poor survival [13, 28–31].

In this study, the prognostic role of FUBP1 and ENO1 tissue expression and the aAb response to them was analyzed in two large cohorts of PDA patients.

## Methods

### Enrollment of patients

The present study was carried out on two different PDA cohorts of patients referred to two different time periods. The first cohort consisted of patients with PDA referred to the Subalpine Hematology Oncology Center, Città della Salute e della Scienza of Turin, enrolled from December 2005 to September 2012, while the second cohort related to the Enoapa multicenter study, active since September 2012 in Piedmont. Inclusion criteria consisted of a new histological or cytological diagnosis of PDA, Eastern Cooperative Oncology Group Performance Status (ECOG-PS) between 0 and 2, absence of previous chemotherapy/radiotherapy treatments and signing of a written informed consent. The exclusion criteria consist of any previous malignant neoplasm with the exception of adequately treated basal cell or squamous cell carcinoma of the skin, carcinoma of the uterine cervix in situ or patients affected by any other malignancies, but disease free for at least 5 years from the date of enrollment and inability to follow-up (1 year). The pre-treatment evaluation is performed by the enrolling center (collection of written informed consent, anamnestic collection, complete physical examination, evaluation of performance status) according to ECOG, demographic information, blood chemistry tests, including CA 19-9, computed tomography abdomen, endoscopic ultrasonography (EUS) and eventually positron emission tomography (PET) in high-risk patients (e.g., high levels of CA19-9) to look for small metastases not otherwise detectable.

The stage of disease assessed at the time of diagnosis, was defined according to the TNM system (UICC, 2017). The main difference between the two cohorts was the different stratification of the recruited patients at baseline, whereby in the Enoapa study subjects were subdivided according to resectable, borderline or locally advanced/metastatic disease, and blood sampling was performed at diagnosis and prior to any treatment option (radical surgery, neoadjuvant chemo-radiotherapy, palliative chemotherapy), whereas in the first cohort, patients that had undergone surgical resection performed the baseline sampling after surgery (not before, as in the Enoapa study). This represented a confounding factor in any comparison between the two populations of surgical patients at baseline.

The clinical data of the patients from the Enoapa cohort were collected and entered into an online database, specifically developed by the Clinical Epidemiology Unit, CPO, University Hospital Città della Salute e della Scienza of Turin.

Of the Enoapa PDA patients, 45 patients that had undergone surgery were analyzed for FUBP1 and ENO1 expression in peritumoral and tumoral tissues fixed in formalin and embedded in paraffin.

### Autoantibody detection

Human recombinant FUBP1 (Tema Ricerca, Castenasco, Italy) and ENO1 (Sigma-Aldrich, Milan, Italy) proteins were coated at 0.5 μg/ml and 2 μg/ml, respectively, in 0.1 mol/l Na_2_CO_3_ at pH 9.6 onto flat-bottomed plates and incubated overnight at 4 °C. Sera of PDA patients were diluted at 1:1000 in PBS containing 1% bovine serum albumin and 0.05% Tween-20 (Sigma-Aldrich, Milan, Italy). Anti-human horseradish peroxidase (Jackson Immuno Research, Cernusco sul Naviglio, Italy) diluted at 1:3000 was then added followed by tetramethylbenzidine (TMB) (Tebu Bio, Magenta, Italy) incubation. Positive and negative controls were incubated as coating and background control. The optical density (OD) delta values were calculated by subtracting the OD of coated wells from the uncoated wells to account for background signal.

### Immunohistochemistry

Formalin-fixed and paraffin-embedded samples of tumor tissue and of the adjacent peri-tumoral pancreas, which were originally collected from the surgically resected specimens for the diagnostic histopathological examination, were retrieved. Histological slides were then cut and stained to evaluate FUBP1 and ENO1 expression by immunohistochemistry. Briefly, peroxidase activity was inhibited by a 3% hydrogen peroxide aqueous solution for 10 min; then, samples were pre-treated by microwave antigen retrieval using EDTA buffer and incubated with FUBP1 (Abcam, Cambridge, UK, diluted at 1:300) or ENO1 antibody (Sigma-Aldrich, diluted at 1:100) for 30 min at room temperature. The rabbit EnVision system (Agilent-Dako, Milan, Italy) or the anti-goat biotinylated followed by streptavidin was used before diaminobenzidine tetrahydrochloride (Agilent-Dako) incubation. Negative and positive controls were included to set up the staining protocol. All slides were stained for the same antigen, together with the same antigen-retrieval buffer and antibody dilution. Tissues were examined in a double-blind fashion, and digital images of representative areas were taken. The presence of positive cells in the tumor and peri-tumoral area of the pancreatic tissues sections was classified as absent (score 0), scarce (score 0.5), moderate (score 1), strong (score 2), or very strong (score 3).

### Statistical analysis

The distribution of the patients’ characteristics was summarized using frequency and percentage for qualitative variables, and media, median and interquartile ranges for continuous variables. Overall survival (OS) was calculated starting from the date of diagnosis to the date of death from any cause or the date of the last follow-up. OS was estimated using the Kaplan–Meier method. The Cox model was used to estimate the hazard ratios (HR) adjusted for the main prognostic factors. Forest plot was used to show HR estimates of subgroup analysis. Logistic models were applied to assess associations between death and its risk indicators. ENO1 and FUBP1 antigens were included as restricted cubic spline [32]. All statistical analyses were performed with Stata version 15 (Stata Corp, College Station). Statistical significance was set at *p* < 0.05.

## Results

### Clinical features of enrolled patients

The first cohort of PDA patients consisted of 186 patients (104 males and 82 females) with a mean age of 65 years. Of these, seven patients did not receive chemotherapy due to rapid clinical progression and were not included in subsequent analyses. Of the remaining 179 patients, 124 received palliative chemotherapy, while 55 underwent radical surgery and received adjuvant chemotherapy (Table 1).

**Table 1:** Clinical characteristics of PDA patient enrolled in the study

	PDA patients		Total
	NON-ENOAPA (cohort 1)	ENOAPA (cohort 2)	
Patients	186	284	470
*Gender, N (%)*			
M	104 (56%)	135 (48%)	239 (51%)
F	82 (44%)	149 (52%)	231 (49%)
Age at diagnosis, mean, median (IQR)	65, 66 (59–72)	66, 67 (60–74)	66, 67 (59–73)
*Year of diagnosis, N (%)*			
2002–2010	147 (79%)	0	147 (31.3%)
2011–2015	39 (21%)	105 (37%)	144 (30.6%)
2016–2020	0	179 (63%)	179 (38.1%)
*Histology, N (%)*			
Adenocarcinoma	181 (97%)	283 (99.6%)	464 (99%)
Other	5 (3%)	1 (0.4%)	6 (1%)
*Disease site, N (%)*			
Head–body	106 (57%)	214 (75%)	320 (68.1%)
Body–tail	78 (42%)	70 (25%)	148 (31.5%)
Missing	2 (1%)	0 (0%)	2 (0.4%)
*Stage of disease, N (%)*			
1	2 (1%)	17 (6%)	19 (4%)
2	49 (27%)	63 (22%)	112 (24%)
3	32 (17%)	74 (26%)	106 (22%)
4	103 (55%)	130 (46%)	233 (50%)
*Metastasis site, N (%)*			
Liver	64 (34%)	41 (15%)	105 (22%)
Lung	12 (7%)	6 (2%)	18 (4%)
Peritoneum	11 (6%)	11 (4%)	22 (5%)
Multiple	34 (18%)	75 (26%)	109 (23%)
No metastases	65 (35%)	151 (53%)	216 (46%)
*Performance status at baseline, N (%)*			
0	108 (58%)	98 (35%)	206 (43.8%)
1	70 (37%)	148 (52%)	218 (46.4%)
2	7 (4%)	31 (11%)	38 (8.1%)
3–4	1 (1%)	7(2%)	8 (1.7%)
*Surgery, N (%)*			
Not performed	120 (64%)	147 (52%)	267 (57%)
Radical	55 (30%)	119 (42%)	174 (37%)
Palliative	11 (6%)	14 (5%)	25 (5%)
Missing	0 (0%)	4 (1%)	4 (1%)
*Chemotherapy, N (%)*			
Adjuvant	55 (29%)	20 (7%)	75 (16%)
Palliative	124 (67%)	123 (43%)	247 (53%)
Not performed	0 (0%)	137 (48%)	137 (29%)
Missing	7 (4%)	4 (2%)	11 (2%)
Ca 19.9 at baseline, mean, median (IQR)	1971.54, 330.0 (61.0–2483.0)	2510.41, 247.5 (22.0–1653.0)	2327.42, 267.0 (31.0–2145.0)
Anti-ENO1 antibodies at baseline, mean, median (IQR)	0.40, 0.29 (0.22–0.44)	0.42, 0.32 (0.19–0.55)	0.41, 0.32 (0.20–0.51)
Anti-FUBP1 antibodies, mean, median (IQR)	0.15, 0.11 (0.06–0.18)	0.21, 0.15 (0.09–0.25)	0.19, 0.12 (0.08–0.22)

A total of 95% of patients had an ECOG-PS of 0–1 at baseline, and 51% had the pancreatic head as site of disease. More than 50% of cases were stage IV, while at diagnosis, the liver accounted for 34% of sites of metastasis, and at baseline about 24% had Gastro Intestinal Cancer Antigen (GICA) values less than or equal to 100U/ml. In 19 patients we were unable to detect ENO1 aAb titers at baseline due to the small amount of serum available.

In the Enoapa cohort there were 284 patients (135 males, 149 females) with a mean age at diagnosis of 66 years. The site of primary disease was the pancreatic head in 65% of cases; metastases were observed in 47% of cases and stage IV disease was observed in 46% of cases. Of all cases, 87% had an ECOG-PS of 0–1, while 52% did not undergo surgical treatment. Ca19.9 at baseline was less than or equal to 100 U/ml in 39% of patients.

### Correlation between FUBP1 and ENO1 tissues expression and circulating aAb levels in the resected PDA patient cohort

The expression of FUBP1 in PDA was significantly upregulated in the TCGA database compared to normal tissues [33]. Moreover, ENO1 overexpression in PDA tissues correlated with tumor progression [34]. The immunohistochemical expression of FUBP1 and ENO1 in selected tumoral and peri-tumoral tissues (*N* = 45) from resected PDA patients was evaluated (Supplementary Fig. 1A–F).

Compared to peri-tumoral tissues or normal pancreases, both ENO1 and FUBP1 were overexpressed in PDA tumors.

As overexpression of tumor-associated antigens (TAA), ENO1 and FUBP1, may break self-tolerance, inducing an immune response against them [35, 36], the presence of circulating aAb to ENO1 and FUBP1 was analyzed in sera from the two cohorts of PDA patients (Table 1). In both cohorts of patients, the levels of ENO1 and FUBP1 aAb were found to be upregulated compared to healthy subjects, similarly to what has been previously observed in a smaller cohort of PDA patients [11] (Supplementary Fig. 1G).

In the cohort of resected patients, the Rho Spearman test showed a significant correlation between circulating aAb and upregulated expression in tumor tissue for FUBP1 (*p* = 0.0268), but not for ENO1 (*p* = 0.3172).

### Circulating aAb to FUBP1 but not to anti-ENO1 correlates with better prognosis in inoperable/advanced stage PDA patients

As circulating aAb to FUBP1 and ENO1 was increased in PDA patients, their prognostic role was evaluated by assessing their levels with the risk of death in the two cohorts, namely cohort 1 and cohort 2, in which resected and non-resected patients were recruited at different times, from 2002 to 2012, and 2012 to 2020, respectively (Table 1).

**Fig. 1:**
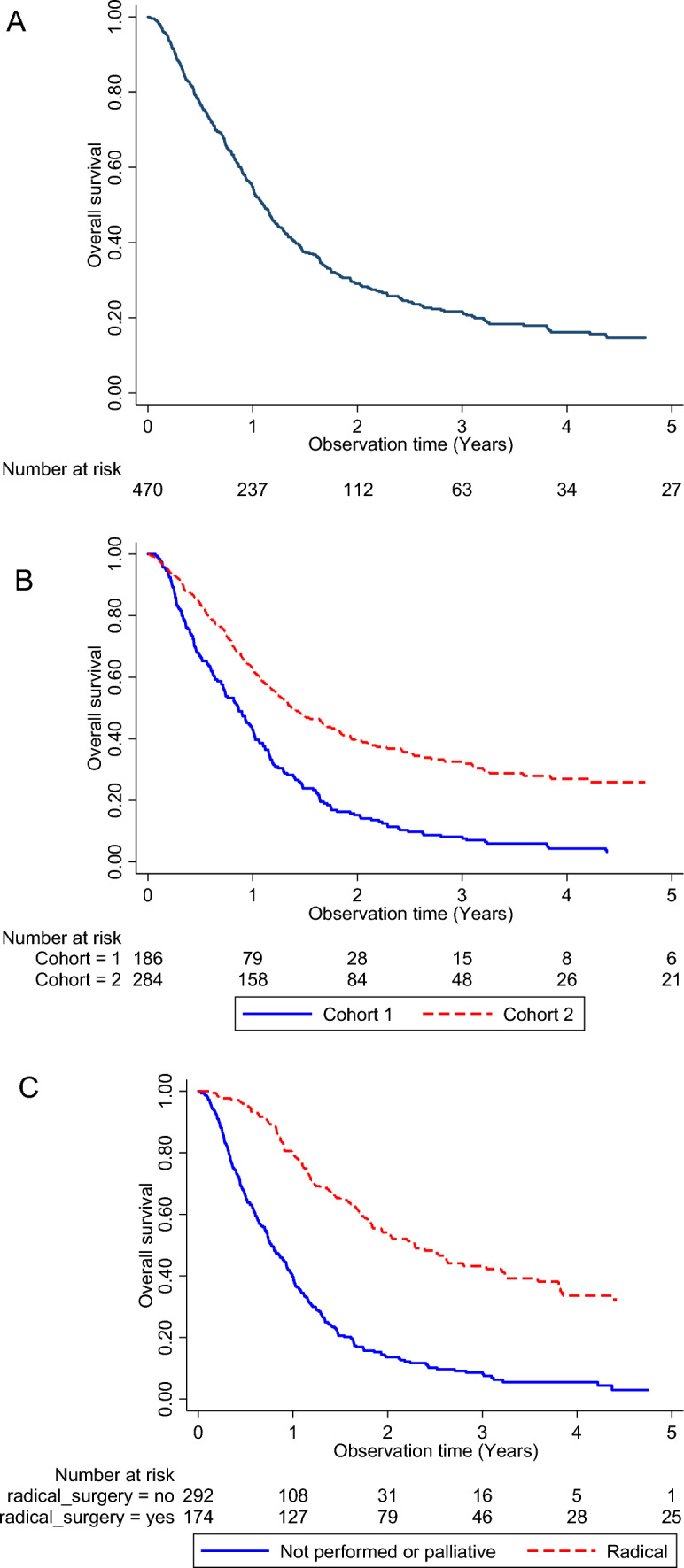
nalysis of OS of PDA patients in the studied cohorts. OS of PDA patients at 5 years from diagnosis (**A**). OS of PDA patients in the older cohort 1 (blue line) and the recent cohort 2 (red dotted line) (**B**). OS of resected PDA patients compared to palliative-treated PDA patients (**C**) (Color figure online)

In both the analyzed cohorts, the 5-year OS was about 15% (Fig. 1A), while the mean OS of cohort 2 was significantly higher than cohort 1 (Fig. 1B). Indeed, resection of the tumor significantly impacted on the OS of PDA patients, as shown in Fig. 1C.

**Fig. 2:**
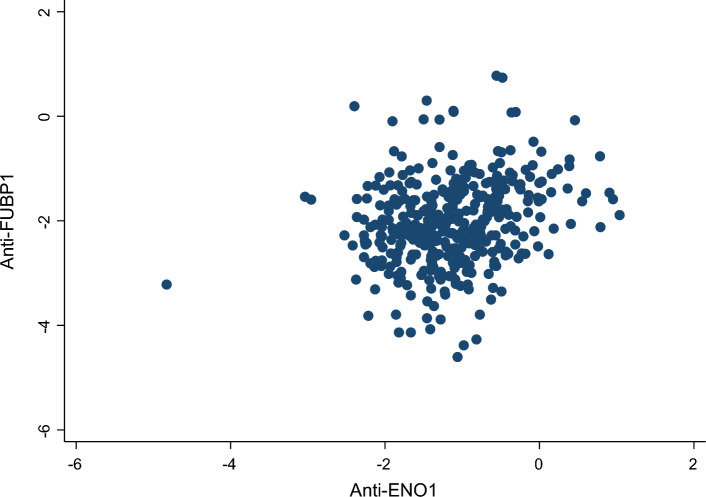
Correlation between logarithmic transformation anti-ENO1 and anti-FUBP1 aAb titers in PDA patients

As surgery can significantly impact the OS of patients, mainly because patients who have undergone surgical resection displayed a lower stage or earlier disease, we subdivided cohort 1 and cohort 2 into surgical and non-surgical patients and measured the correlation between anti-FUBP1 and anti-ENO1 aAb levels and risk of death at 12 months from diagnosis. As surgical patients tend to survive more than patients who do not undergo surgery, this allows better prediction of the prognostic value of the aAb to the two TAA. As shown in Fig. 2, there is a low positive correlation between anti-FUBP1 and anti-ENO1 aAb titers (Pearson’s *r* = 0.25, *p* < 0.001), indicating that the two antibodies are independent factors.

A Cox regression model was employed to analyze other variables that can affect OS of PDA patients (Table 2). Levels of aAb to FUBP1 and ENO1 were subdivided into tertiles (low, intermediate and high). Performance status (*p* < 0.001), Ca19.9 (*p* = 0.017), high anti-ENO1 (*p* = 0.016) and intermediate anti-FUBP1 (*p* = 0.013) aAb levels were negative prognostic factors.

**Table 2:** Estimation of the predictors of OS for all cases using the Cox model

	Crude HR (CI) *N* = 470	*p* value	Adjusted HR (CI) *N* = 346	*p* value
Age (continuous)	0.99 (0.98–1.01)	0.516	1.00 (0.99–1.02)	0.944
*Performance status*				
1 versus 0	1.09 (0.88–1.37)	0.424	1.92 (1.45–2.56)	0.000
2–4 versus 0	1.75 (1.24–2.48)	0.002	2.32 (1.46–3.69)	0.000
*Surgery* [*4 missing*]				
Radical versus (palliative or not performed)	0.30 (0.23–0.38)	0.000	0.33 (0.25–0.44)	0.000
Baseline Ca19.9 × 10^3^(continuous) [43 missing]	1.03 (1.01–1.05)	0.002	1.03 (1.01–1.06)	0.017
Disease site (body-head vs. tail-body) [2 missing]	0.51 (0.41–0.64)	0.000	0.67 (0.51–0.88)	0.005
Cohort (2 vs. 1)	0.48 (0.39–0.59)	0.000	0.50 (0.38–0.66)	0.000
*Anti-ENO1 antibody titer at baseline* [57 *missing*]				
(0.243–0.405) versus ≤ 0.243	1.22 (0.93–1.59)	0.148	1.26 (0.93–1.71)	0.134
> 0.405 versus ≤ 0.243	1.29 (0.99–1.69)	0.058	1.45 (1.07–1.98)	0.016
*Anti-FUBP1 antibody titer* [71 *missing*]				
(0.092–0.177) versus ≤ 0.092	1.17 (0.90–1.54)	0.247	1.47 (1.08–2.00)	0.013
> 0.177 versus ≤ 0.092	1.07 (0.81–1.40)	0.641	1.15 (0.83–1.57)	0.403

Radical surgery was associated with a greater OS (*p* < 0.001). Cohort 2 displayed a high OS compared to cohort 1 (*p* < 0.001)

### High levels of circulating anti-FUBP1 aAb are a good prognostic marker of tail-body pancreatic cancer.

**Fig. 3:**
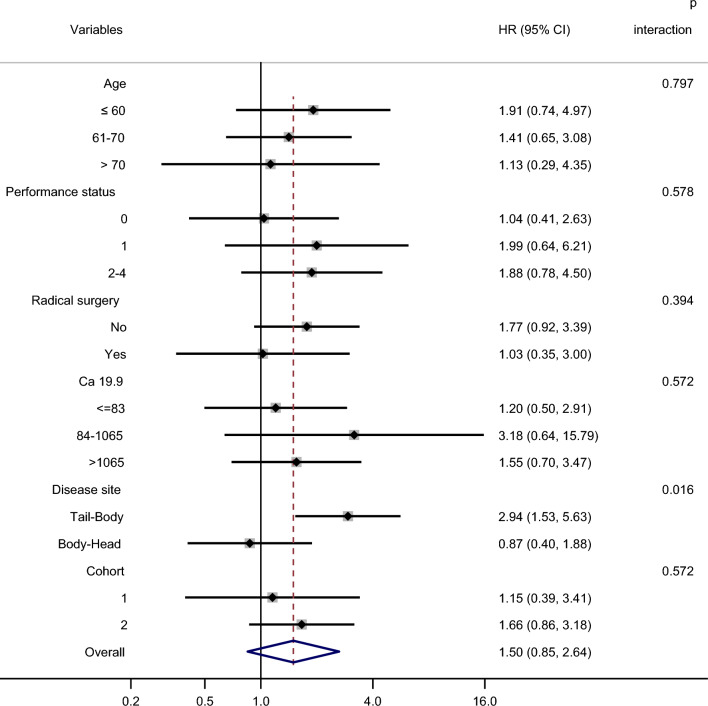
Forest plot for subgroup analysis of anti-FUBP1 aAb levels on OS

**Fig. 4:**
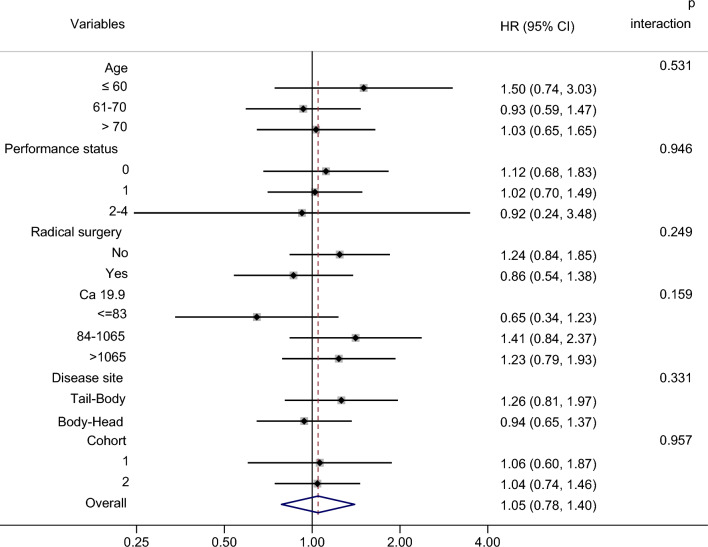
Forest plot for subgroup analysis of anti-ENO1 aAb levels on OS

The results of subgroup analysis of anti-FUBP1 or anti-ENO1 aAb titer for OS are shown with forest plots (Figs. 3 and 4).

The overall HRs of circulating aAb levels to both FUBP1 and ENO1 were not statistically significant (with *p* values of 1.50 and 1.05, respectively). However, the significant interaction *p*-value (0.016) between values and disease site indicates that anti-FUBP1 aAb level is a good prognostic marker when the tumor is in the tail body of the pancreas. Notably, the other variables did not influence the prognostic effect of anti-FUBP1 aAb levels, suggesting that this marker is independent.

### FUBP1 autoantibody titer correlates with prognosis in non-resected PDA patients.

**Fig. 5:**
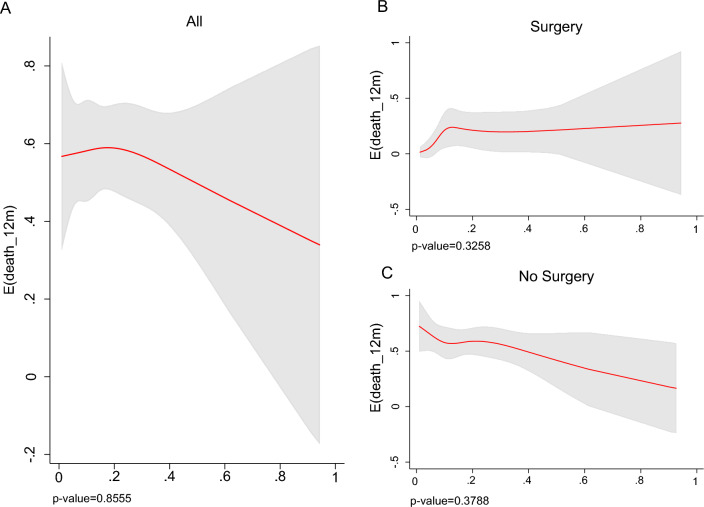
Spline of anti-FUBP1 aAb titer and expected 12-month mortality in all (**A**), resected (**B**) and non-resected (**C**) PDA patients (*p*-value for test of linearity is shown)

**Fig. 6:**
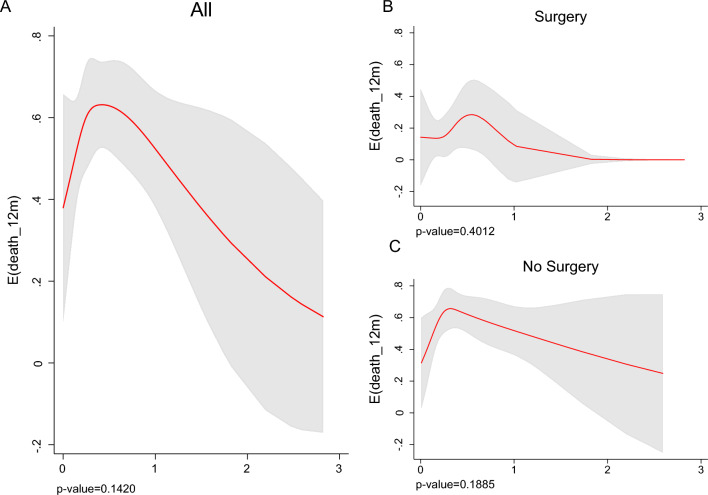
Spline of anti-ENO1 aAb level and expected 12-month mortality in all (**A**), resected (**B**) and non-resected (**C**) PDA patients (*p*-value for test of linearity is shown)

We used the spline tool to test the prognostic values of anti-TAA aAb and the risk of death in PDA patients. Although no threshold values of antibody titers were identified, the curve spline slopes allowed correlation of the anti-TAA aAb titer with risk of death (Figs. 5 and 6).

Analyzing 12-month mortality, the relationship between anti-FUBP1 aAb levels and prognosis is evident for non-resected PDA patients with elevated aAb OD values. In patients who had undergone radical surgery, there was no relationship between FUBP1 aAb levels and the risk of mortality.

Anti-ENO1 aAb levels showed no relationship with mortality risk when PDA patients were resected, but there was a relationship for high titer of ENO1 aAb when patients did not undergo surgery.

Considering the head body of the pancreas as the tumor site (Supplementary Figs. 2, 3), a slight relationship of both FUBP1 and ENO1 aAb titer with the prognosis was observed. If the tumor was located in the body tail, there was no relationship with FUBP1 aAb levels, while there was a correlation with elevated ENO1 antibody values at this site.

## Discussion

PDA is predicted to become the second leading cause of cancer-related mortality within the next decade, with limited effective treatment options and a dismal long-term prognosis for patients [1]**.** For this reason, it is urgently required to implement effective therapeutic strategies and identify biomarkers for early diagnosis and stratification of PDA patient treatment.

The present study considers two distinct cohorts of patients recruited in different period of time (2005–2012 and 2012–2022) and shows that a considerable progress has been made over the years in the field of surgery, chemotherapy regimens and diagnostic capability, to increase overall survival of PDA patients (Fig. 1A–C), in agreement with previous observation [37–41].

Many non-autoimmune diseases such as cancer are characterized by aAb responses to TAA [42–44]. By employing a serological proteome approach, we found circulating aAb against TAA in sera of most PDA patient [9, 10]. In addition, chemotherapy treatment increased circulating aAb not only to ENO1, but to other TAA also, including FUBP1 [11].

Here we investigated the prognostic role of both anti-ENO1 and FUBP1 expression in tissues and aAb in PDA patients. We found that the presence of both circulating aAb to ENO1 and FUBP1, but not their combination, is independent prognostic factors of PDA prognosis. High anti-ENO1 and intermediate anti-FUBP1 aAb levels are negative prognostic factors, whereas an increased level of anti-FUBP1 aAb is a good prognostic factor for PDA tumors in the pancreas tail body only. This is a very important finding, suggesting that the increased levels of anti-FUBP1 aAb can be used as a prognostic biomarker in clinical practice. Overall, the data obtained in this study indicate that levels of aAb to ENO1 and FUBP1 might provide useful medical information, not only for the prognosis of the disease, but also for the site of onset of PDA, allowing a better outcome of currently available therapies.

An higher expression of both TAA in PDA and—for the first time—a positive correlation between high FUBP1 expression in tumors and circulating anti-FUBP1 aAb in resected PDA patients was observed. This indicates that high levels of anti-FUBP1 aAb mirror its high expression in PDA and prompted us to hypothesize that monitoring anti-FUBP1 aAb can be used to evaluate changes in FUBP1 tumor tissue expression and eventually target FUBP1 to reduce tumor burden [43].

Unlike FUBP1, the correlation between high levels of circulating aAb to ENO1 and its high tissue expression was not observed due to high dispersion of the ENO1 tissue expression score values. We cannot rule out the possibility of low ENO1 tumor expression in patients with high ENO1 aAb is a consequence of the immune-mediated elimination of high ENO1-expressing tumor cells, and selection of ENO1 negative, less antigenic tumor clones [45].

We previously demonstrated that there is a positive correlation between circulating aAb to a phosphorylated isoform of ENO1 and survival of PDA patients [10]. By contrast, in this study, we observed a negative correlation between aAb to ENO1 and OS. However, aAb to phosphorylated ENO1 was found in PDA patients [10], whereas ENO1 is overexpressed in PDA tissues, but can also be present on the surface of bacteria, and ENO1 aAb is also induced in healthy subjects [10, 46]. In the case of aAb to phosphorylated ENO1, we cannot distinguish aAb that are induced by ENO1 overexpression in tumor tissues or by bacterial infection.

We have shown that DNA vaccination against TAA effectively delays tumor progression in a genetically engineered mouse PDA model [11, 46], which can be translated to PDA patient clinical management. Therefore, we hypothesized that high circulating levels of aAb to ENO1 [10] or FUBP1 may correlate with increased OS, underlying a specific anti-tumor immunity against these TAA, which may help identify patients eligible for immunotherapy based on ENO1 or FUBP1 DNA vaccination, even in combination with chemotherapy.

## Conclusion

PDA has a dismal prognosis due to the lack of effective treatments and diagnostic and prognostic biomarkers. One main prognostic biomarker is Ca 19.9, although it is not specific for PDA. Novel prognostic biomarkers are needed for more effective prediction of the outcome of disease and the response to therapy.

The evaluation of circulating aAb to ENO1 and FUBP1 can be useful tool to predict the outcome in PDA patients.

### Supplementary Information

Below is the link to the electronic supplementary material.

Supplementary file1 (DOCX 6182 kb)

## Data Availability

The data generated in this study are available upon request from the corresponding authors.

## Abbreviations

ENO1: Alpha-enolase
FUBP1: Far upstream element-binding protein 1
PDA: Pancreatic ductal adenocarcinoma
aAb: Autoantibodies
OD: Optical density
CA 19.9: Carbohydrate antigen 19.9
HR: Hazard ratios
OS: Overall survival
PS: Performance status scale

## References

[1] Sivapalan L, Kocher HM, Ross-Adams H (2022). The molecular landscape of pancreatic ductal adenocarcinoma. Pancreatology.

[2] Malafa PM (2015). Defining borderline resectable pancreatic cancer: emerging consensus for an old challenge. J Natl Compr Cancer Netw.

[3] Pereira SP, Oldfield L, Ney A (2020). Early detection of pancreatic cancer. Lancet Gastroenterol Hepatol.

[4] Wang Z, Li Y, Ahmad A (2011). Pancreatic cancer: understanding and overcoming chemoresistance. Nat Rev Gastroenterol Hepatol.

[5] Zhang X, Li J, Wang Y (2022). A diagnostic model with igm autoantibodies and carcinoembryonic antigen for early detection of lung adenocarcinoma. Front Immunol.

[6] Patel AJ, Tan TM, Richter AG (2022). A highly predictive autoantibody-based biomarker panel for prognosis in early-stage NSCLC with potential therapeutic implications. Br J Cancer.

[7] Almaguel FA, Sanchez TW, Ortiz-Hernandez GL (2021). Alpha-enolase: emerging tumor-associated antigen, cancer biomarker, and oncotherapeutic target. Front Genet.

[8] Rastogi A, Ali A, Tan SH (2016). Autoantibodies against oncogenic ERG protein in prostate cancer: potential use in diagnosis and prognosis in a panel with C-MYC. AMACR HERV-K Gag G Cancer.

[9] Tomaino B, Cappello P, Capello M (2007). Autoantibody signature in human ductal pancreatic adenocarcinoma. J Prot Res.

[10] Tomaino B, Cappello P, Capello M (2011). Circulating autoantibodies to phosphorylated α-enolase are a hallmark of pancreatic cancer. J Prot Res.

[11] Mandili G, Curcio C, Bulfamante S (2020). In pancreatic cancer, chemotherapy increases antitumor responses to tumor-associated antigens and potentiates DNA vaccination. J Immunother Cancer.

[12] Duan J, Bao X, Ma X (2017). Upregulation of far upstream element-binding protein 1 (fubp1) promotes tumor proliferation and tumorigenesis of clear cell renal cell carcinoma. PLoS ONE.

[13] Chen Y, Liu J, Geng N (2020). Upregulation of far upstream element-binding protein 1 (fubp1) promotes tumor proliferation and unfavorable prognosis in tongue squamous cell carcinoma. Int J Biol Markers.

[14] Huang Y, Xu X, Ji L (2016). Expression of far upstream element binding protein 1 in B cell non Hodgkin lymphoma is correlated with tumor growth and cell adhesion mediated drug resistance. Mol Med Rep.

[15] Wang S, Wang Y, Li S (2022). Far upstream element -binding protein 1 (FUBP1) participates in the malignant process and glycolysis of colon cancer cells by combining with c-Myc. Bioengineered.

[16] Marquina G, Manzano A, Casado A (2018). Targeted agents in cervical cancer: beyond bevacizumab. Curr Oncol Rep.

[17] Douillard JY, Oliner KS, Siena S (2013). Panitumumab-FOLFOX4 treatment and ras mutations in colorectal cancer. N Engl J Med.

[18] Debaize L, Troadec MB (2019). The master regulator fubp1: its emerging role in normal cell function and malignant development. Cell Mol Life Sci.

[19] Zhang XX, Chen H, Li HY (2020). Long non-coding RNA small nucleolar RNA host gene 6 aggravates pancreatic cancer through upregulation of far upstream element binding protein 1 by sponging microrna-26a-5p. Chin Med J.

[20] Jang M, Park BC, Kang S (2009). Far upstream element-binding protein-1, a novel caspase substrate, acts as a cross-talker between apoptosis and the c-myc oncogene. Oncogene.

[21] Avigan MI, Strober B, Levens D (1990). A far upstream element stimulates c-myc expression in undifferentiated leukemia cells. J Biol Chem.

[22] Duncan R, Bazar L, Michelotti G (1994). A sequence-specific, single-strand binding protein activates the far upstream element of c-myc and defines a new DNA- binding motif. Genes Dev.

[23] Chan AK-Y, Pang JC-S, Chung NY-F (2014). Loss of CIC and FUBP1 expressions are potential markers of shorter time to recurrence in oligodendroglial tumors. Mod Pathol.

[24] Kang M, Kim HJ, Kim T-J (2020). Multiple functions of Fubp1 in cell cycle progression and cell survival. Cells.

[25] Zhao Y, Hu X, Liu Y (2017). ROS signaling under metabolic stress: cross-talk between AMPK and AKT pathway. Mol Cancer.

[26] Yokoyama C, Sueyoshi Y, Ema M (2017). Induction of oxidative stress by anticancer drugs in the presence and absence of cells. Oncol Lett.

[27] Liu J, Chung HJ, Vogt M (2011). JTV1 co-activates FBP to induce USP29 transcription and stabilize p53 in response to oxidative stress. EMBO J.

[28] Fan P, Ma J, Jin X (2018). Far upstream element-binding protein 1 is up-regulated in pancreatic cancer and modulates immune response by increasing programmed death ligand 1. Biochem Biophys Res Comun.

[29] Zhang Y, Chen J, Zhou N (2021). FUBP1 mediates the growth and metastasis through TGF*β*/Smad signaling in pancreatic adenocarcinoma. Int J Mol Med.

[30] Xu W, Yang W, Wu C (2021). Enolase 1 correlated with cancer progression and immune-infiltrating in multiple cancer types: a pan-cancer analysis. Front Oncol.

[31] Hoang AT, Vizio B, Chiusa L (2021). Impact of tissue enolase 1 protein overexpression in esophageal cancer progression. Int J Med Sci.

[32] Harrell FE (2001). Regression modeling strategies: with applications to linear models, logistic regression, and survival analysis.

[33] Livak KJ, Schmittgen TD (2001). Analysis of relative gene expression data using real-time quantitative PCR and the 2(-delta delta C(T)) method. Methods.

[34] Yin H, Wang L, Liu H-L (2018). ENO1 overexpression in pancreatic cancer patients and its clinical and diagnostic significance. Gastroenterol Res Pract.

[35] Pupa SM, Invernizzi AM, Forti S (2001). Prevention of spontaneous neu-expressing mammary tumor development in mice transgenic for rat proto-neu by DNA vaccination. Gene Ther.

[36] Lu Y, Wei YQ, Tian L (2003). Immunogene therapy of tumors with vaccine based on xenogeneic epidermal growth factor receptor. J Immunol.

[37] Ghaneh P, Palmer D, Cicconi S (2023). Immediate surgery compared with short-course neoadjuvant gemcitabine plus capecitabine, FOLFIRINOX, or chemoradiotherapy in patients with borderline resectable pancreatic cancer (ESPAC5): a four-arm, multicentre, randomised, phase 2 trial. Lancet Gastroenterol Hepatol..

[38] Sugawara T, Rodriguez Franco S, Sherman S (2023). Association of adjuvant chemotherapy in patients with resected pancreatic adenocarcinoma after multiagent neoadjuvant chemotherapy. JAMA Oncol.

[39] Maruta A, Iwashita T, Yoshida K (2021). Evaluation of preoperative diagnostic methods for resectable pancreatic cancer: a diagnostic capability and impact on the prognosis of endoscopic ultrasound-guided fine needle aspiration. BMC Gastroenterol.

[40] Takagi K, Umeda Y, Yoshida R (2023). Role of surgery for pancreatic ductal adenocarcinoma in the era of multidisciplinary treatment. J Clin Med.

[41] Springfeld C, Ferrone CR, Katz MHG (2023). Neoadjuvant therapy for pancreatic cancer. Nat Rev Clin Oncol.

[42] Ying X, Han S-X, He C-C (2017). Autoantibodies against glucose-regulated protein 78 as serological biomarkers in metastatic and recurrent hepatocellular carcinoma. Oncotarget.

[43] Khageh Hosseini S, Kolterer S, Steiner M (2017). Camptothecin and its analog SN-38, the active metabolite of irinotecan, inhibit binding of the transcriptional regulator and oncoprotein FUBP1 to its DNA target sequence FUSE. Biochem Pharmacol.

[44] Niccolai E, Cappello P, Taddei A (2016). Peripheral ENO1-specific T cells mirror the intratumoral immune response and their presence is a potential prognostic factor for pancreatic adenocarcinoma. Int J Oncol.

[45] Dunn GP, Old LJ, Schreiber RD (2004). The three Es of cancer immunoediting. Annu Rev Immunol.

[46] Cappello P, Rolla S, Chiarle R (2013). Vaccination with ENO1 DNA prolongs survival of genetically engineered mice with pancreatic cancer. Gastroenterology.

