# Supplementary material for: Circulating autoantibodies to alpha-enolase (ENO1) and far upstream element-binding protein 1 (FUBP1) are negative prognostic factors for pancreatic cancer patient survival
Source: Clin Exp Med. 2023 Nov 1;23(8):5089–100. doi: 10.1007/s10238-023-01236-5 (PMC10725354; doi:10.1007/s10238-023-01236-5)
Supplement: Supplementary file 1 — Supplementary file1 (DOCX 6182 kb) [file 10238_2023_1236_MOESM1_ESM.docx]

**Supplementary Figure 1.** FUBP1 and ENO1 expression in peritumoral and tumoral pancreatic tissues and circulating FUBP1 and ENO1 aAb in sera of both cohorts of PDA patients. FUBP1 and ENO1 immunohistochemical staining of peritumoral and tumoral pancreases in a cohort of resected patients. Graphs report the FUBP1 (A) and ENO1 (D) mean values ± SD expression in peritumoral and tumoral tissues. Statistical significance is shown. Representative images of peritumoral (B-E) and tumoral (C-F) pancreatic tissues. Each section was counterstained with hematoxylin. Original magnification 10X.ELISA detection of aAb to FUBP1 and ENO1 in PDA patients (G). White circles indicate values of aAb in Cohort 1 (n=171), while black circles indicate values of aAb in Cohort 2 (n=246). Red lines indicate the median.

A

B

C

**Supplementary Figure 2.** Spline of FUBP1 auto-antibody levels and expected 12-month mortality in all patients (A) and in those with head-body (B) and body-tail (C) disease sites; (p-value for test of linearity).

C

B

A

**Supplementary Figure 3.** Spline of ENO1 auto-antibody levels and expected 12-month mortality in all patients (A) and in those with head-body (B) and body-tail (C) disease sites (p-value for test of linearity).
